# Therapeutic activity of sarpogrelate and dopamine D_2_ receptor agonists on cardiovascular and renal systems in rats with alloxan-induced diabetes

**DOI:** 10.1186/s40360-021-00526-6

**Published:** 2021-10-26

**Authors:** Mohammed Ahmed Fouad Shalaby, Hekma A. Abd El Latif, Mohamed El Yamani, May Ahmed Galal, Sherifa Kamal, Ikhlas Sindi, Raneem Masaood

**Affiliations:** 1 Pharmaceutical Sciences Department, Pharmacy Programme, Batterjee Medical College, 21442 Jeddah, Saudi Arabia KSA; 2 Pharmacology and Toxicology Department, Faculty of Pharmacy, Cairo University, Cairo, Egypt; 3 Pharmacology Department, National Organization for Drug Control and Research, Giza, Egypt; 4 Research Unit, Batterjee Medical College, KSA Jeddah, Saudi Arabia

**Keywords:** Cardiovascular, Diabetic nephropathy, Myocardial injury, TNFα1, Dopamine receptors

## Abstract

**Background:**

Dopamine D_2_ receptor agonists, bromocriptine and cabergoline, are notable medications in the treatment of Parkinsonism, hyperprolactinemia, and hyperglycemia. An affiliation was found between the initiation of myocardial injury ailment and long term treatment with dopamine D_2_ agonist drugs identified with the partial activation of 5-hydroxytryptamine receptor 2 A (5-HT2A). The investigation aimed to examine the activity of sarpogrelate (a 5-HT2A receptor blocker) in reducing myocardial injury prompted by extended haul utilisation of D_2_ receptor agonists in rats with alloxan-induced diabetes.

**Methods:**

Both bromocriptine and cabergoline were managed independently and combined with sarpogrelate for about a month in diabetic nephropathy rats. Both tail-cuff blood pressure and the BGL were recorded weekly. For all animals, the kidney hypertrophy index, serum creatinine, blood urea nitrogen, alanine transaminase, and aspartate transaminase levels were measured after one month of treatment. The severity of the cardiac injury was assessed by the estimation of lactate dehydrogenase-1 (LDH-1), cardiac troponin I, and tumor necrosis factor alpha 1 (TNF1). The triphenyltetrazolium chloride (TTC) staining method was used to determine the experimental myocardial infarction (MI) size.

**Results:**

Bromocriptine and cabergoline created a significant reduction in BGL, BP, and kidney hypertrophy index in diabetic nephropathy rats. Administration of bromocriptine and cabergoline, alone, or in combination with sarpogrelate fundamentally diminished the blood concentrations of alkaline phosphatase (ALP), Aspartate aminotransferase (AST), urea, and creatinine. Bromocriptine and cabergoline alone showed a noteworthy increase in the LDH-1, Troponin I, and TNF1 levels in the serum (p < 0.05). Paradoxically, utilising bromocriptine or cabergoline with sarpogrelate treatment altogether decreased the levels of the myocardial biomarkers in the serum. A mix of bromocriptine or cabergoline with sarpogrelate diminished the level of the myocardial infarct size in the heart assessed through the TTC staining method.

**Conclusions:**

The examination demonstrated that the combined use of sarpogrelate with bromocriptine or cabergoline decreased the potential adverse effects of these two drugs on the myocardial tissues.

## Background

Among the various diabetes progress complications, diabetic nephropathy is the foremost well-known renal concern and the ultimate source of end-stage renal disease. The later phases of diabetic nephropathy define glomerulosclerosis. It is represented by transforming growth factor-ß1 overexpression, extracellular matrix deposition, and glomerular structure loss [1]. Hyperglycemia is the primary factor for diabetic nephropathy progression [2]. Several in vitro studies of streptozotocin-induced diabetic nephropathy demonstrated that renal damage is claimed to occur due to the excessive production of reactive oxygen species (ROS) under hyperglycemic conditions [3–5]. Bromocriptine is a sympatholytic dopamine D2 agonist that has been approved for treating type 2 diabetes without the risk of hypoglycemia because there is no insulin secretion. Supported previous studies stated bromocriptine administration is believed to strengthen low hypothalamic dopamine levels and inhibit excessive sympathetic tone within the central system, leading to a reduction in the post-meal plasma glucose levels because of the enhanced suppression of hepatic glucose production [6, 7]. Agonists working on dopamine D_2_ receptors can lower hypertension signs by the inhibition of sodium–potassium adenosine triphosphatase (Na/k ATPase) activity, vasodilation, and sympathetic nerve activity inhibition. Bromocriptine may protect against the ischemia-reperfusion (I/R) injury of the kidney utilising p44/42 mitogen-initiated protein kinase actuation [8] and prevent chronic nephropathy [9].

Conversely, the bromocriptine-actuated hypotension was wholly nullified by pretreatment with metoclopramide, a dopamine D_2_ receptor antagonist that crosses the blood brain barrier [10, 11]. However, the use of D_2_ agonists is often related to cardiovascular complications, including hypotension and myocardial disorder. There is an identified relationship between the utilisation of D_2_-like R agonists in patients with Parkinson’s disease and cardiopathy, especially in the early phase of therapy [12]. D_2_ receptors are pleiotropic receptors that have multiple effects, in that, activating them inhibit adenylyl cyclase, resulting in the inhibition of voltage-gated calcium ions and activation of potassium conductance [13]. Early studies suggested that the use of D_2_ receptor agonists results in a reduction in pituitary tumour mass [14].

It was demonstrated that long term use of bromocriptine therapy was associated with an increased risk of developing heart disease, which occurred in a cumulative dose-dependent treatment [15]. Dopamine D_2_ receptor agonists are classified as ergot dopamine D_2_ agonists and non-ergot D2 agonists. Bromocriptine and cabergoline (ergot derivative) are related to valvularcardiopathy since these two drugs have agonistic activities against both dopamine D_2_ and serotonin 5-HT2A receptors. Pramipexole (non-ergot derivative) shows few incidences of the onset of heart disease since it does not affect 5-HT2B receptors [16–18]. Both sarpogrelate and ketanserin (selective 5-HT2A/2B antagonists) reduce cardiac fracture, infarct size, and changes within the electrocardiogram resulting from myocardial injury. Likewise, these outcomes bolster the view that serotonin and 5-HT2A may augment the harmful impacts of ischemic injury within the heart [19, 20]. Sarpogrelate was found to possess beneficial impacts in peripheral vascular sickness, restenosis after coronary stenting, pneumonic hypertension, intense and relentless myocardial localised necrosis [21]. The current study aimed to identify the possible protective effect of sarpogrelate, a 5-HT2A receptor blocker, associated with the cumulative use of D_2_ receptor agonist drug therapy on cardiac and renal functions in a rat model of diabetic nephropathy.

## Methods

### Materials

Sarpogrelate was obtained from Shanghai Linebon Ltd., Shanghai, China. Bromocriptine was sourced from Novartis, Italy. Cabergoline was purchased from Pfizer, Italy. Domperidone was purchased from Jamjoom Pharmaceuticals Co., KSA. Alloxan was obtained from Sigma-Aldrich (St Louis, MO, USA). Urea, creatinine, alkaline phosphatase, aspartate aminotransferase activity, LDH-1 assay kits, and the TNF alpha ELISA kit were obtained from Abcam, USA. The troponin I test kit was obtained from Encode Medical Engineering Company, Jeddah, KSA. 2,3,5-Triphenyltetrazolium chloride was obtained from Gold Biotechnology, USA.

### Animals

Forty-two Wistar albino male rats 5–7 weeks of age weighing 150–200 g were obtained from the animal house of Batterjee Medical College, Jeddah, KSA. Three rats were housed per cage under controlled standard laboratory conditions in monitored ventilated cages and spontaneously given food and water. The ethical committee of research at Batterjee Medical College approved the steps of the investigation. Tutelage was taken, particularly with relevant housing conditions, to avoid or minimise the animals’ discomfort. The animals were kept on solid floored cages with a deep layer of sawdust, and the cages were changed daily. Data were coded before analysis so that the treatment group could not be identified before the analysis was completed. At the end of the study, all animals were euthanised by thiopental (intravenous injection, 150 mg/kg) for tissue collection.

### Induction of diabetes by alloxan

Alloxan monohydrate was dissolved in sterile normal saline. Diabetes was induced in 30 rats (150–200 g) by a single intraperitoneal injection of alloxan (5 %) 150 mg/kg b.w. The rats were kept in a fasted state for 12 h before being injected with alloxan. The fasting plasma glucose was measured in blood samples collected from the tail vein. The rats that showed a plasma glucose level of 200 mg/dl or more were considered diabetic and taken in the study [22].

### Experimental design

Tests took place four weeks after the induction of diabetes. The diabetic rats were divided randomly into six diabetic groups and one healthy control group, each group comprising six rats. The samples were calculated based on the resource equation method [23]. Once there was a stable rise in the urea and creatinine levels in the blood, drugs were injected once daily for one month.

1. The normal control group (Saline, IP).
2. Diabetic control group (Saline, IP).
3. Diabetic group treated with bromocriptine (4 mg/kg b.w, IP) [24].
4. Diabetic group treated with cabergoline (0.6 mg/kg b.w ,IP) [25].
5. Diabetic group treated with sarpogrelate (50 mg/kg b.w, IP) [26].
6. Diabetic group treated with a combination of bromocriptine and sarpogrelate at the same doses.
7. Diabetic group treated with a combination of cabergoline and sarpogrelate at the same doses.

Dopamine D_2_ agonists’ bromocriptine and cabergolin were used to induce myocardial infarction in the rats. The drugs were administered daily doses at 4 mg/kg and 0.6 mg/kg IP respectively in two groups of rats for one month. 50 mg/kg IP of sarpogrelate in combination with bromocriptine and cabergoline respectively was administered daily in two groups of rats for one month for testing its potential protective effect on the heart.

### Determination of blood glucose level

Blood glucose levels were tested on the7th, 14th, 21th, and 28th days from the beginning of the treatment. One millimetre of tail end was cut and a drop of blood was used for blood glucose tests using an advanced glucometer (Roche, USA). The accuracy of the glucometer was checked with the O-toluidine method [27].

### Blood pressure recording

Basal blood pressure was measured by employing the tail-cuff non-invasive blood pressure recording apparatus (Ugobasile instruments, Italy). The mean systolic blood pressure was measured for each group of rats once time weekly for one month.

Each rat was placed in an exceeding restrainer, and an appropriate cuff sensor was mounted on its tail and warmed to about 33–35 °C. The tail cuff was inflated to a pressure above 200 mmHg. Systolic blood pressure and diastolic blood pressure was measured directly by the tail-cuff and pulse sensor two hours after treatment with drugs [28].

### Estimation of liver and kidney functions

At the end of the study, a blood sample was withdrawn through the retro-orbital venous plexus method, kept at 37 °C for 30 min, and centrifuged at 4 °C, 3000 rpm for 10 min. Then, the separated serum was stored at -20 °C for various biochemical analyses. Alkaline phosphatase and aspartate aminotransferase activities were determined using the method described by King and King [29]. The procedure of Tietz et al. [30] was used to determine the serum creatinine concentration, while the serum urea concentration was determined by the method given by Kaplan [31]. At the end of the study, all the rats were euthanized. The kidney samples were collected, washed with normal saline, and weighed. The kidney hypertrophy index was calculated by determining the kidney weight and body weight ratio (g/g) x10^3^.

### Estimation of serum biomarkers of myocardial injury

The severity of the cardiac injury was assessed by estimating the lactate dehydrogenase (LDH-1) and cardiac troponin I (cTnI) levels in serum. The LDH-1 and cTnI levels were analysed by spectrophotometric methods using commercially available diagnostic kits, consistent with the methods of Nieland [32].

### Serum TNF-αconcentration

The serum TNF-alpha cytokines level was identified by the ELISA technique using a quantitative sandwich enzyme immunoassay technique (Abcam Company). The test was done according to the company’s instructions. The ELISA reader’s optical density at 405 nm was immediately calculated and applied on a standard curve to sort out the cytokine concentration.

### Evaluation of myocardial infarct size by TTC

For assessment of the myocardial infarcted area, the hearts were removed, washed in phosphate-buffered saline, frozen, and stored at -20 °C. The frozen hearts were sliced into 1 mm sections along the long axis, from apex to base. Triphenyltetrazolium chloride (TTC) staining was used to assess the myocardial tissue viability and determine the myocardial infarction size. The tissue slices were incubated in 1 % TTC phosphate-buffered saline solution, pH 7.4, at 37 °C for 20 min. Tissues were fixed in 10 % PBS-buffered formalin overnight at 2–8 °C. Each side of every TTC-stained tissue slice was photographed with the photographic Optika camera (Digi, Italy) to differentiate the red-stained viable and white-unstained necrotic tissues [33]. Digital photographs were downloaded to a computer. Areas stained in white and red were measured using the SigmaScan software (SPSS Science) in trace-measurement mode. That mode was used to measure either the ischemic or infarcted areas, which may be a sum of calibrated pixels during a defined region. This was done manually by drawing an image layer on the photograph [34]. The infarction size percentage was calculated using the following equation:

$$\mathrm{The}\;\mathrm{percentage}\;\mathrm{of}\;\mathrm{infarct}\;\mathrm{volume}\;=\;\mathrm{Infarct}\;\mathrm{volume}\;/\;\mathrm{Total}\;\mathrm{volume}\;\mathrm{of}\;\mathrm{slice}\;\times\;100$$

### Statistical analysis

The data and statistical analysis done complied with the recommendations for experimental design and analysis in pharmacology [35]. The results were expressed as mean ± SE. The significance of the differences between the values was performed by a one-way ANOVA test and Tukey Kramer’s Multiple Comparison Test using the GraphPad Prism 9 software. P < 0.05 was considered to be a significant difference.

## Results

### Estimation of blood glucose levels

The diabetic control group showed a significant increase in the BGL compared to the healthy control group. On repeated administration of the bromocriptine (4 mg/kg) and cabergoline (0.6 mg/kg) individually or in combination with sarbogrelate, a significant (*p* < 0.05) decrease was observed in blood glucose by time, compared to the diabetic control group throughout the four weeks of treatment (Table 1).

**Table 1:** Effect of the tested drugs on BGL in alloxan-induced diabetic rats

BGL mg/dL				
Groups	Week 1	Week 2	Week 3	Week 4
Normal control group	136.5 ± 18.52	121.33 ± 12.08	119.25 ± 11.35	109 ± 10.92
Diabetic control group	359.75 ± 45.13*	328 ± 141.34*	428.5 ± 121.17*	396.5 ± 80.60*
Diabetic group treated with bromocriptine	191 ± 16.89^#^	199.25 ± 65.05^#^	238.25 ± 56.88^#^	284.25 ± 148.00^#^
Diabetic group treated with Cabergoline	213.75 ± 37.74^#^	211.75 ± 38.62^#^	293 ± 213.15^#^	280.25 ± 220.48^#^
Diabetic group treated with sarpogrelate	326.25 ± 93.45	298.25 ± 122.37	393.75 ± 118.77	369.75 ± 43.15
Diabetic group treated with bromocriptine + sarpogrelate	247.75 ± 50.35^#^	215.5 ± 33.20^#^	310.25 ± 70.59^#^	288.25 ± 109.41^#^
Diabetic group treated with cabergoline + sarpogrelate	196 ± 54.20^#^	158.75 ± 35.01^#^	235.5 ± 182.22^#^	215.75 ± 141.46^#^

Values shown are means ± SEM; *n* = 6 rats per group. * *P* < 0.05, significantly different from the normal control group; # *P* < 0.05, significantly different from the diabetic control group

### Kidney hypertrophy index

The results showed that the kidney hypertrophy index significantly increased in the diabetic group of rats compared with the normal control rats. However, the index was markedly reduced by both bromocriptine and cabergoline treatments, individually or mixed with sarpogrelate (as seen in Table 2). There was no effect of using sarpogrelate on the diabetic kidney index.

**Table 2:** Effect of the tested drugs on Kidney Hypertrophic Index in alloxan-induced diabetic rats

Kidney Hypertrophic Index	
Groups	KHI ( g/g * 1000 )
Normal control group	4.555 ± 0.423
Diabetic control group	6.6125 ± 0.57*
Diabetic group treated with bromocriptine	4.7475 ± 0.33^#^
Diabetic group treated with cabergoline	4.81 ± 0.49^#^
Diabetic group treated with sarpogrelate	5.90 ± 0.27
Diabetic group treated with bromocriptine + sarpogrelate	4.715 ± 0.61^#^
Diabetic group treated with cabergoline + sarpogrelate	4.46 ± 0.48^#^

Values shown are means ± SEM; *n* = 6 rats per group. * *P* < 0.05, significantly different from the normal control group; # *P* < 0.05, significantly different from the diabetic control group

### Hemodynamic parameter (Antihypertensive activity)

Alloxan-induced diabetes in rats showed a significant rise in blood pressure after three weeks from the induction of diabetes. Daily oral administration of bromocriptine and cabergoline, individually or in combination with sarpogrelate showed a significant decrease in the systolic blood pressure in the third and fourth weeks of treatment (Table 3). There was no effect of sarpogrelate on the BP in diabetic rats.

**Table 3:** Effect of the tested drugs on mean blood pressure in alloxan-induced diabetic rats

Mean Blood Pressure MBP (mmHg)				
Groups	Week 1	Week 2	Week 3	Week 4
Normal control group	110 ± 4.01	118.25 ± 8.99	108.75 ± 4.57	111.5 ± 4.20
Diabetic control group	112.5 ± 9.14	121.75 ± 11.35	150.75 ± 5.25*	151.25 ± 5.12*
Diabetic group treated with bromocriptine	115 ± 9.12	102.75 ± 6.8	109 ± 10.23^#^	121.75 ± 15.26^#^
Diabetic group treated with cabergoline	103.75 ± 7.0	105.5 ± 9.14	123 ± 7.87^#^	107.75 ± 107.75^#^
Diabetic group treated with sarpogrelate	118.75 ± 10.87	114.75 ± 8.53	137.75 ± 8.53	139 ± 18.56
Diabetic group treated with bromocriptine + sarpogrelate	104.5 ± 7.59	104.75 ± 4.99	106.25 ± 11.08^#^	118 ± 13.03^#^
Diabetic group treated with cabergoline + sarpogrelate	109.25 ± 11.58	105 ± 9.05	106 ± 18.3^#^	104.25 ± 5.5^#^

Values shown are means ± SEM; *n* = 6 rats per group. * *P* < 0.05, significantly different from the normal control group; # *P* < 0.05, significantly different from the diabetic control group

### Estimation of liver and kidney functions

The serum levels of AST, ALP, urea, and creatinine were recorded as the indicators of liver and kidney functions, as presented in Table 4. Data revealed that the diabetic control group had a significant increase in the previous biomarkers’ serum concentrations compared to the healthy control rats. The daily administration of bromocriptine and cabergoline, both alone or in combination with sarbogrelate, significantly decreased the serum concentrations of the previous biomarkers compared to the positive control of diabetic rats. There was no marked effect of sarpogrelate treatment on the previous biochemical indicators in the diabetic rats.

**Table 4:** Effect of the tested drugs on the liver and kidney functions in alloxan-induced diabetic rats after 4 weeks

Biochemicals				
Group	ALP(IU/L)	AST(IU/L)	Urea (mmol/L)	Creatinine (mmol/L)
Normal control group	60.85 ± 8.77	25.04 ± 4.96	43.38 ± 4.77	0.46 ± 0.07
Diabetic control group	89.05 ± 10.44*	45.86 ± 7.57*	75.81 ± 18.85*	1.89 ± 0.19*
Diabetic group treated with Bromocriptine	68.83 ± 7.27^#^	34.18 ± 5.78^#^	60.41 ± 6.22^#^	1.04 ± 0.26^#^
Diabetic group treated with Cabergoline	73.27 ± 7.93^#^	21.37 ± 10.05^#^	52.09 ± 2.23^#^	0.79 ± 0.11^#^
Diabetic group treated with Sarpogrelate	82.16 ± 23.83	41.47 ± 10.02	69.41 ± 7.65	1.68 ± 0.25
Diabetic group treated with Bromocriptine + sarpogrelate	64.8 ± 7.21^#^	31.03 ± 6.44^#^	44.4 ± 8.23^#^	1.26 ± 0.25^#^
Diabetic group treated with cabergoline + sarpogrelate	73.44 ± 6.68^#^	33.79 ± 11.18^#^	51.16 ± 10.43^#^	1.33 ± 0.21^#^

Values shown are means ± SEM; *n* = 6 rats per group. * *P* < 0.05, significantly different from the normal control group; # *P* < 0.05, significantly different from the diabetic control group

### Myocardial biomarkers

Animals treated with bromocriptine and cabergoline for one month in doses 10 mg/kg and 0.6 mg/kg, respectively, displayed significant elevation of the LDH-1 activity in the serum. In contrast, it was observed that using a combination of bromocriptine and cabergoline with sarpogrelate treatment significantly decreased the level of the biomarkers in the serum. The results in Table 5 indicate that the quantitative test of the troponin I reagent kit showed only positive results with the bromocriptine and cabergoline treated groups. The results of the ELISA test suggest that the expression levels of TNF-alpha 1 in the diabetic rat groups treated with bromocriptine or cabergoline individually were significantly higher compared to the diabetic control rats. The groups administered a combination of bromocriptine and cabergoline with sarbogrelate showed lower TNF-alpha 1 expression levels than the groups treated with bromocriptine or cabergoline individually (Table 5).

**Table 5:** Effect of the tested drugs on the Lactate dehydrogenase-1, Troponin I, and TNFα1 in alloxan-induced diabetic rats

Myocardial biomarkers			
Groups	LDH-1 (IU/L)	Troponin I (pg/ml)	TNFα1 (pg/mL)
Normal control group	17.42 ± 0.69	92.12 ± 9.19	06.12 ± 0.45
Diabetic control group	18.50 ± 1.53	95.73 ± 8.23	08.32 ± 0.56
Diabetic group treated with bromocriptine	40.50 ± 10.69^#^	313.43 ± 29.43^#^	36.56 ± 2.32^#^
Diabetic group treated with cabergoline	36.86 ± 12.47^#^	143.23 ± 12.93^#^	31.20 ± 4.23^#^
Diabetic group treated with carpogrelate	19.55 ± 2.20	101.17 ± 8.45	09.74 ± 0.98
Diabetic group treated with bromocriptine + sarpogrelate	27.75 ± 3.91 ^a^	241.34 ± 17.83^a^	14.84 ± 1.23 ^a^
Diabetic group treated with cabergoline + sarpogrelate	34.57 ± 6.60 ^b^	128.83 ± 13.53	08.59 ± 0.76 ^b^

Values shown are means ± SEM; *n* = 6 rats per group. # *P* < 0.05, significantly different from the diabetic control group. a *P* < 0.05, significantly different from the diabetic group treated with bromocriptine. b *P* < 0.05, significantly different from the diabetic group treated with cabergoline

## Evaluation of myocardial injury

**Fig. 1:**
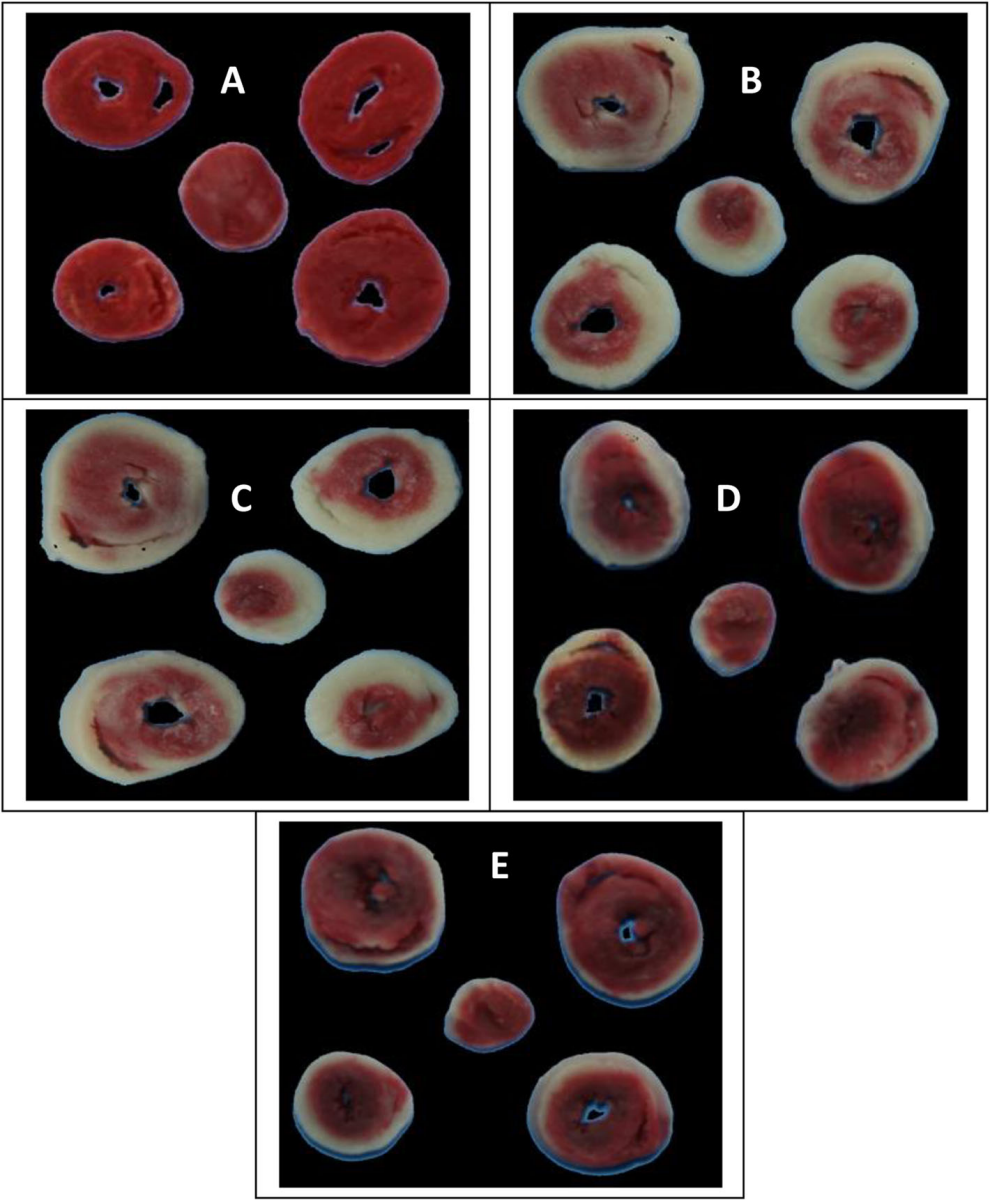
Effect of the tested drugs on the hearts in alloxan-induced diabetic rats after four weeks of treatment. Triphenyl tetrazolium chloride stained cross-section of the heart of **A** Diabetic control rat showing uniform staining pattern. Both **B** bromocriptine treated group, and **C** cabergoline treated group showing a relative large pale, TTC-negative area of infarct. Both **D** Bromocriptine + sarbogrelate treated group, and **E** Cabergoline + sarbogrelate treated group showing a relative small pale, TTC-negative area of infarct

**Fig. 2:**
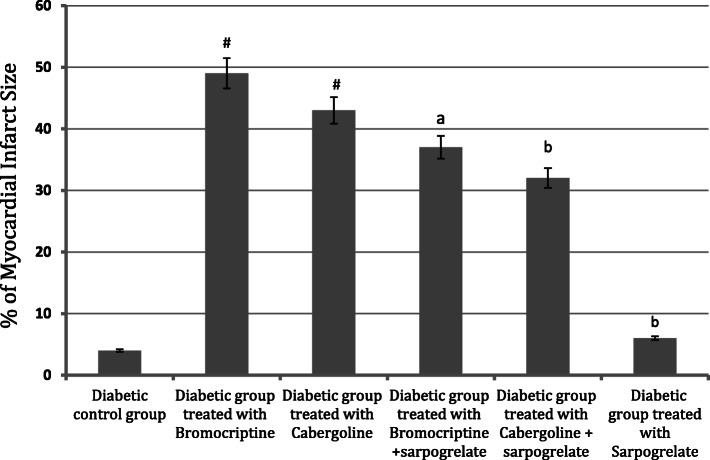
Effect of the tested drugs on the percentage of myocardial infarct size in alloxan-induced diabetic rats. Values shown are mean ± SEM; *n* = 6 rats per group. # *P* < 0.05, significantly different from the diabetic control group. (a) *P* < 0.05, significantly different from the diabetic group treated with bromocriptine. (b) *P* < 0.05, significantly different from the diabetic group treated with cabergoline

The myocardial infarction size was used as an indicator of the progress of the myocardial injury. As demonstrated in Fig. 1, TTC unstained areas correlated closely with areas of infarction, the infarction areas of the myocardial sections for the treated groups were compared to the diabetic control group. The hearts of rats treated with bromocriptine (4 mg/kg) and cabergoline (0.6 mg/kg) showed a significant increase in risk area infarction. In contrast, the combination of bromocriptine or cabergoline with sarpogrelate (50 mg/kg) reduced the myocardial infarct size (Fig. 1). The infarction sizes in bromocriptine and cabergoline treated groups 48.80±6.15%, and 42.21±5.21% resp. were larger than bromocriptine and cabergoline in combination with sarpogrelate treated groups 36.23±4.90%, and 31.22±3.46% resp. (Fig. 2).

## Discussion

Both bromocriptine and cabergoline induced hypoglycemic activity, which may have been due to enhanced suppressive hepatic glucose production [36]. The exact mechanism of their action as antidiabetic substances is not entirely identified. Bromocriptine decreases the hepatic production of glucose, increases glucose transporter production, or increases or mimics glucagon-like peptide-1 activity [37]. Its contribution to hypoglycaemia may be because it modulates the neurotransmitter action in the brain and has been shown to improve glucose tolerance and insulin resistance in animal models of obesity and diabetes [38]. Bromocriptine was approved by the Food and Drug Administration (FDA) in May 2009 for the treatment of type 2 diabetes.

In the present study, diabetic nephropathy was the most common cause of renal complication and the leading of hypertension. Both bromocriptine and cabergoline showed a marked antihypertensive activity; this result agrees with some previous reports [39, 40]. The antihypertensive activity is related to the activation of dopamine D_2_ receptors, which can lower the blood pressure by inhibition of Na/k ATPase activity, vasodilation, and inhibition of the sympathetic nerve activity. Both bromocriptine and cabergoline induced a marked improvement in kidney function by decreasing urea and creatinine serum levels. The pharmacological pathways that explain this effect have not been sufficiently determined. The result agrees with some of the previous studies, suggesting that the development of therapies directed to increase renal D_2_ receptor expression and function may provide novel and practical approaches to treating renal injury [41].

The noted severe adverse effect of bromocriptine and cabergoline on the heart was represented in myocardial injury and infarction, approved in the present study by using relative overdoses for a month of treatment. The action may be related to the agonistic properties of dopamine D_2_ and serotonin 5-HT2A receptors, increasing the heart pumping rate. It was reported that both bromocriptine and cabergoline (ergot derivative) have been associated with heart disease since the two drugs have both dopamine D_2_ and serotonin 5-HT2A receptors agonistic properties [17, 18]. It is hypothesised that the 5-HT2A receptor, which is a common contributing factor underlying aspects of vasoconstriction and cardiovascular disorders [42] and vasodilation through activating nitric oxide (NO) synthase (NOS) via serotonin 5-HT1B receptors in endothelial cells, possesses different effects on regulating vascular tone. These facts lead to the consideration that sarpogrelate, a 5-HT2A receptor blocker may increase coronary blood flow via either attenuation of vasoconstriction through the 5-HT2A receptor blockade or of vasodilation by the relative stimulation of NOS through 5-HT1B receptor [43].

According to the current observed data, the combination of bromocriptine and cabergoline with the new chemical agent sarpogrelate (selective 5-HT2A/2B antagonists) decreased the adverse effects of these two drugs on the heart. The protective effects of sarpogrelate on myocardial tissue were approved by its ability to decrease the secretion of myocardial biomarkers as shown, by decreasing LDH-1, Troponin I, and TNF alpha 1 during the treatment of bromocriptine and cabergoline. Sarpogrelate attenuates cardiac dysfunction and infarct size; these results support the view that serotonin and 5-HT2A might contribute to the harmful effects of ischemic injury in the heart. Regarding the biochemical study, sarpogrelate can be considered a safe drug for liver and kidney functions.

## Conclusions

According to the given study, the dopamine D_2_ receptor agonists bromocriptine and cabergoline induced myocardial infarction. However, using sarpogrelate in combination with bromocriptine or cabergoline decreased their potential adverse effects on the myocardial tissues. Thus, we suggest sarpogrelate deserves additional experimental and clinical research related to cardiovascular diseases.

## Data Availability

The datasets used and analysed during the current study are available from the corresponding author on reasonable request.

## Abbreviations

ALT: Alanine aminotransferase
AST: Aspartate aminotransferase
TNF: Tumour necrosis factor
TTC: Triphenyltetrazolium chloride
LDH: Lactate dehydrogenase
TTC: Triphenyltetrazolium chloride
5-HT: 5-hydroxytryptamine
ELISA: Enzyme-linked immunosorbent assay
